# Comprehensive Atlas of the Myelin Basic Protein Interaction Landscape

**DOI:** 10.3390/biom11111628

**Published:** 2021-11-03

**Authors:** Evgeniya V. Smirnova, Tatiana V. Rakitina, Rustam H. Ziganshin, Georgij P. Arapidi, George A. Saratov, Anna A. Kudriaeva, Alexey A. Belogurov

**Affiliations:** 1 Shemyakin-Ovchinnikov Institute of Bioorganic Chemistry, Russian Academy of Sciences, 117997 Moscow, Russia; smirnova.evgeniya@gmail.com (E.V.S.); taniarakitina@yahoo.com (T.V.R.); ziganshin@mail.ru (R.H.Z.); arapidi@gmail.com (G.P.A.); saratovgosha@gmail.com (G.A.S.); anna.kudriaeva@gmail.com (A.A.K.); 2 Federal Research and Clinical Center of Physical-Chemical Medicine of Federal Medical Biological Agency, 119435 Moscow, Russia; 3 Moscow Institute of Physics and Technology, National Research University, 141701 Dolgoprudny, Moscow Region, Russia; 4 Department of Fundamental Medicine, Lomonosov Moscow State University, 117192 Moscow, Russia

**Keywords:** myelin basic protein, MBP, interactome, formaldehyde cross-linking, MS-based proteomic analysis

## Abstract

Intrinsically disordered myelin basic protein (MBP) is one of the key autoantigens in autoimmune neurodegeneration and multiple sclerosis particularly. MBP is highly positively charged and lacks distinct structure in solution and therefore its intracellular partners are still mostly enigmatic. Here we used combination of formaldehyde-induced cross-linking followed by immunoprecipitation and liquid chromatography-tandem mass spectrometry (LC-MS/MS) to elucidate the interaction network of MBP in mammalian cells and provide the list of potential MBP interacting proteins. Our data suggest that the largest group of MBP-interacting proteins belongs to cellular proteins involved in the protein translation machinery, as well as in the spatial and temporal regulation of translation. MBP interacts with core ribosomal proteins, RNA helicase Ddx28 and RNA-binding proteins STAU1, TDP-43, ADAR-1 and hnRNP A0, which are involved in various stages of RNA biogenesis and processing, including specific maintaining MBP-coding mRNA. Among MBP partners we identified CTNND1, which has previously been shown to be necessary for myelinating Schwann cells for cell-cell interactions and the formation of a normal myelin sheath. MBP binds proteins MAGEB2/D2 associated with neurotrophin receptor p75NTR, involved in pathways that promote neuronal survival and neuronal death. Finally, we observed that MBP interacts with RNF40–a component of heterotetrameric Rnf40/Rnf20 E3 ligase complex, recruited by Egr2, which is the central transcriptional regulator of peripheral myelination. Concluding, our data suggest that MBP may be more actively involved in myelination not only as a main building block but also as a self-regulating element.

## 1. Introduction

Myelinization of the axons is critical for the rapid conduction of the nerve impulses. Myelin is maintained as a tightly packed, lipid-rich multilayer membrane envelope to function as an electrical insulator. The importance of the myelin membrane is evident in terms of demyelinating diseases such as multiple sclerosis, which lead to severe neurological disability. Myelin is produced by specialized glial cells in both, central and peripheral nervous systems (CNS and PNS, respectively). Genetic abnormalities in myelin genes or an autoimmune destruction of myelin lead to severe neurological damage. Plenty of the lesions developed during dys- or demyelination processes can be associated with the partial violation of the tight interactions of myelin proteins with lipid bilayer. Myelin basic protein (MBP) is one of the most abundant among myelin proteins. It presents in high amount in CNS and PNS in oligodendrocytes and Schwann cells, respectively [1,2]. MBP is a peripheral membrane-associated semisubmersible protein with highly disordered structure in an aqueous solution [3,4,5]. However, number of data indicate that the interaction of MBP with ligands triggers the formation of secondary structures and some degree of MBP folding into a more compacted scaffold [6]. Aggarwal et al. showed that MBP controls biogenesis of myelin by weak forces emerging from MBP’s inherent phase separation ability [7]. The interaction of MBP with the inner part of the membrane bilayer leads to charge neutralization and triggers its self-association into larger polymers and a phase transition to a dense protein network. The MBP interaction network has common features with the formation of amyloid fibrils [7]. This process occurs through phenylalanine-mediated hydrophobic and amyloid-like interactions, which provide the molecular basis for protein extrusion and “fastening” of myelin membranes. Data from Aggarwal et al. reveal the physico-chemical mechanism of how the cytosolic protein controls the morphology of complex membrane architecture [7]. Their results provide an explanation for the key mechanism of myelin membrane biogenesis with the possibility of using these data to suppress demyelinating diseases of the central nervous system.

MBP has many isoforms resulting from both alternative splicing and miscellaneous post-translational modifications [8]. It is present in the nervous system as isomers with different charge, and it is evident that charge-mediated effects modulate its function and interactions with ligands such as membrane surfaces [9,10,11,12]. Recently it was shown that MBP may be hydrolyzed by proteasome without ubiquitination [13] through novel class of Basic Elementary Autonomous Degrons (BEADs) interacting with PA28-capped proteasomes [14]. MBP is one of the major myelin sheath autoantigens in multiple sclerosis (MS) and animal models of autoimmune neurological disorders [15,16]. It is recognized [17] and cleaved [18,19] by autoantibodies and is efficient substrate for the immunoproteasome [20]. Post-translational modifications of MBP may play an important role in the pathogenesis of MS [21,22]. Removal of arginine occurs at several sites and has been increased in multiple sclerosis [21], and the degree of removal (or citrullination) of MBP correlates with the severity of multiple sclerosis [23]. In the study by Wang et al., recombinant murine analogs of two isoforms of the classic 18.5 kDa MBP, natural C1 and C8 charge isoforms (rmC1 and rmC2, respectively), were used as model proteins to elucidate the structure and function of charge isomers [24]. A number of biochemical and biophysical methods have been used to study the differences between the two isoforms in terms of structure and interaction with ligands, including calmodulin (CaM), various detergents, nucleotide analogs and lipids. In general, the results indicate that rmC8 in comparison with rmC1 is insufficient both in its structure and especially in function. Although the binding properties of CaM are very similar between the two forms, their interactions with membrane mimics were different. In addition, using fluorescently labeled nucleotides, MBP was observed to bind to ATP and GTP, but not AMP; and this binding of nucleoside triphosphates was inhibited in the presence of CaM [24]. Recently it was shown that CaM may protect MBP from ubiquitin-independent proteasomal hydrolysis [25], which contributes to the development of autoimmune neurodegeneration [26].

Summarizing, elucidation of the mechanisms that guide myelin membrane biogenesis may result not only in deeper understanding of the pathogenesis of the autoimmune neurodegeneration, but also in the novel therapeutic approaches. Here using combination of formaldehyde-induced cross-linking followed by immunoprecipitation and liquid chromatography-tandem mass spectrometry (LC-MS/MS) we showed that MBP interacts with eight major proteomic groups related to: (i) protein synthesis machinery; (ii) mitochondrial proteins, (iii) mRNA splicing, transport and maintenance, (iv) reorganization of the actin cytoskeleton at the plasma membrane and cell adhesion, (v) cytoskeleton and intracellular traffic, (vi) ubiquitin-proteasome system, (vii) protein quality control, and (viii) chromatin remodeling.

## 2. Materials and Methods

### 2.1. Cells and Transfection

HEK 293T cells were obtained from the Russian Cell Culture Collection (RCCC, Institute of Cytology of the Russian Academy of Sciences, St-Petersburg, Russia). HEK 293T cells were maintained by passage in Dulbecco’s modified Eagle’s medium supplemented with 100 μg/mL streptomycin, 100 units/mL penicillin, and 10% fetal bovine serum (FBS) (pH 7.2–7.4) in a humidified atmosphere containing 5% CO_2_ at 37 °C. HEK293T cells were transfected with the pBud4.1EF_MBPwt_flag_strep [14] using Lipofectamine LTX Reagent with PLUS Reagent (Thermo Fisher Scientific, Waltham, MA, USA) according to the manufacturer’s instructions. All the experiments were conducted at 48 h after transfection.

### 2.2. Formaldehyde Cross-Linking

The stock 4% formaldehyde solution was obtained by dissolving paraformaldehyde in water for 2 h at ∼80 °C. The resulting solution was passed through the 0.22-micron filter and stored in the dark at room temperature (RT) for maximum of 4 weeks. The working formaldehyde solution was obtained by appropriate diluting the stock solution in PBS. For cross-linking, transfected HEK 293 cells were detached with 0.25% trypsin-EDTA solution, transferred into 15 mL reaction tube, collected in a by centrifugation and washed 3 times with PBS. Finally, the pellet was resuspended in 1 mL of working solution of formaldehyde. Cells were incubated for 7 min with gentle agitation at RT and then collected by centrifugation at 1800× *g* for 3 min (RT) which gave in total 10 min exposure to formaldehyde. The supernatant was discarded, and cells were resuspended in 0.5 mL ice-cold solution of 1.25 M glycine in PBS to quench the cross-linking reaction. Cell pellet was washed again with glycine in PBS and lysed in 1 mL TNE buffer (50 mM Tris HCl, pH 8.0, 150 mM sodium chloride, 1% NP40, 1 mM EDTA) supplemented with protease inhibitors (Merk, Burlington, MA, USA) and 1 mM PMSF for 30 min on ice. After 30 min, cell lysates were sonicated with an ultrasonic homogenizer, treated with 2.5 units of DNAse I (Thermo Fisher Scientific, Waltham, MA, USA) and sonicated again. To remove insoluble debris, lysates were centrifuged for 20 min at 10,000× *g* and 4 °C, and the supernatants were passed through the 0.22-micron syringe filters. The cleared lysates were immediately used for the immunoprecipitation. 1% of the cleared lysates was kept as input controls.

### 2.3. Immunoprecipitation

The cleared lysates were incubated with 20 μL of anti-FLAG M2 Affinity Gel (Sigma-Aldrich, St. Louis MO, USA) or Pierce Protein A/G Agarose (Thermo Fisher Scientific, Waltham, MA, USA) slurry at 4 °C for overnight. Following incubation, agarose beads with immunocomplexes were washed with TNE buffer five times, and immunocomplexes were eluted from agarose beads with sample buffer (65.8 mM Tris HCl, pH 6.8, 10% glycerol, 1% SDS, 0.01% bromophenol blue) at 65 °C for 5 min. The supernatants were treated with 5 μL of 2-mercaptoethanol at 65 °C for 5 min. Supernatants containing immunocomplexes were resolved by sodium dodecyl sulfate–polyacrylamide gel electrophoresis (SDS-PAGE) and obtained gels were stained using Coomassie blue.

### 2.4. Mass Spectrometry Analysis

The strips from the bands stained by Coomassie blue were excised and subjected to a trypsin in-gel digestion procedure. In-gel digestion of protein with trypsin was performed as described previously [27]. After overnight tryptic digestion, the resulting peptides were extracted from the gel blocks. Samples were loaded to in house-made trap column (20 × 0.1 mm), packed with Inertsil ODS3 3 μm sorbent (GL Sciences, Tokyo, Japan), in the loading mobile phase (2% acetonitrile (ACN), 98% H_2_O, 0.1% TFA) at flow rate 10 μL/min and separated in a in house-made [28] fused-silica column (300 × 0.1 mm) packed with Reprosil PUR C18AQ 1.9 (Dr. Maisch, Ammerbuch, Germany) at RT into an emitter made using P2000 Laser Puller (Sutter Instrument, Novato, CA, USA). Reverse-phase chromatography was performed using an Ultimate 3000 Nano LC System (Thermo Fisher Scientific, Waltham, MA, USA), which was connected to the Orbitrap Q Exactive Plus mass spectrometer (Thermo Fisher Scientific, Waltham, MA, USA) via a nanoelectrospray source (Thermo Fisher Scientific, Waltham, MA, USA). As mobile phase A, water containing 0.1% (*v/v*) formamide was used and, as mobile phase B, acetonitrile containing 0.1% formamide (*v/v*), 20% water (*v/v*) was used. Peptides were eluted from the trap column with a linear gradient: 3–6% of B for 3 min; 6–25% of B for 30 min, 25–40% of B for 25 min, 40–60% of B for 4 min, 60% of B for 3 min, 60–99% of B for 0.1 min, 99% B during 10 min, 99–2%B for 0.1 min at 500 nL/min flow rate. After each gradient run, the column was preequilibrated with the buffer A for 10 min. MS data was collected in DDA mode. MS1 parameters were the following: resolution—70 K, scan range—350–1500, max injection time—30 s, AGC target—3 × 10^6^. Ions were isolated with 1.4 *m/z* window, preferred peptide match and isotope exclusion. Dynamic exclusion was set to 30 s. MS2 fragmentation was performed in HCD mode at 17.5 K resolution with normalized collision energy (NCE) of 29%, max injection time of 50 s, AGC target—2 × 10^5^, loop count—10. Other settings were the following: charge exclusion—unassigned, 1, >7. The mass spectrometry proteomics data have been deposited to the ProteomeXchange Consortium via the PRIDE [29,30] partner repository with the dataset identifier PXD028685 and 10.6019/PXD028685.

### 2.5. Data Analysis

Raw LC-MS/MS data from the Orbitrap Q Exactive Plus mass spectrometer were converted to mgf peak lists with MSConvert software (ProteoWizard Software Foundation). The following parameters were used for this procedure, “--mgf --filter peakPicking true [1,2]”. For an exhaustive protein identification, obtained peak lists were processed by MASCOT (version 2.5.1, Matrix Science Ltd., London, UK) and X! Tandem (ALANINE, 1 February 2017, The Global Proteome Machine Organization) against the UniProt Knowledgebase, taxon human, (http://www.uniprot.org, accessed on 12 May 2021) with the concatenated reverse decoy dataset. The precursor and fragment mass tolerance were set at 20 ppm and 50 ppm, respectively. The database search parameters were settled as follows: tryptic digestion with one possible missed cleavage, static modification for carbamidomethyl (C), and dynamic/flexible modifications for oxidation (M). Selected parameters for X! Tandem allowed to rapidly detect protein N-terminal acetylation, peptide N-terminal glutamine ammonia loss or peptide N-terminal glutamic acid water loss. The resulting files were subjected to Scaffold 4 (version 4.2.1, Proteome Software Inc, Portland, OR, USA) for validation and further analysis.

## 3. Results and Discussion

**Figure 1:**
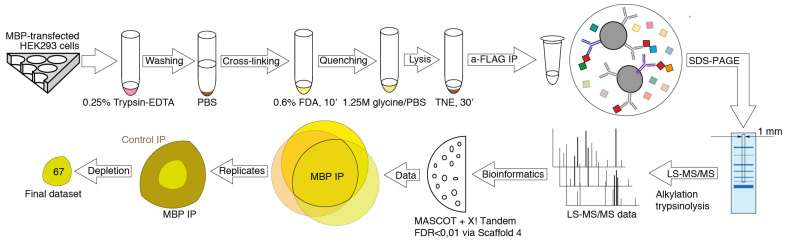
Experiment pipeline. HEK293T cells overexpressing Flag-tagged human MBP were detached, washed, and exposed to formaldehyde. The formaldehyde treated cells were washed and lysed in the mild conditions on ice. Protein complexes containing MBP were immunoprecipitated from the lysates using anti-Flag-agarose. The immunoprecipitated material was resolved by gradient SDS-PAGE. The cross-linked immunoprecipitated complexes were detected by Coomassie Blue staining. The 1 mm wide strips from the middle of each lane were cut and subjected to the in-gel-trypsinolysis procedure and mass spectrometry-based proteomics analysis. A total of three experiments were carried out with triplicate repeats in each.

To elucidate potential binding partners of MBP, we performed formaldehyde crosslinking, immunoprecipitation and mass spectrometry analysis of cell lysates of HEK293T cells overexpressing Flag-tagged human MBP (Figure 1). There are several reasons why we used HEK293T cells overexpressing Flag-tagged MBP: (i) There are only a few immortalized cell lines mimicking oligodendrocytes. Despite expression of surface markers similar to those of oligodendrocytes, they still do not fully represent native cells. (ii) Usage of primary oligodendroglial cultures is not obvious due to the heterogenicity and troubles with either transfection or transduction procedures. Finally, amount of endogenous MBP in native oligodendrocytes is dramatically high therefore it may effectively compete with recombinant tagged MBP, displacing it from protein complexes. (iii) Commercially available antibodies partially cross-react with eukaryotic proteome thus generating false-positive results. Additionally, usage of such low-affinity antibodies requires mild washing conditions in contrast to highly specific and affine anti-FLAG antibodies.

**Figure 2:**
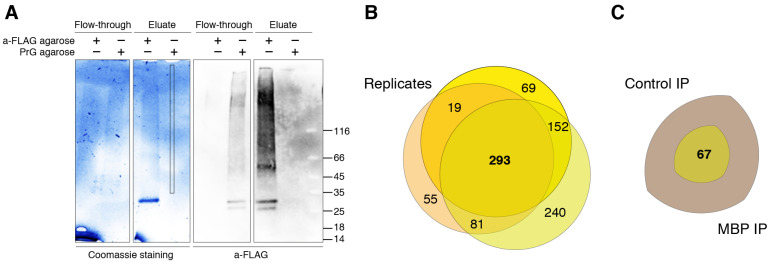
MBP immunoprecipitation and LS-MS/MS data. (**A**) Crosslinked MBP immunocomplexes resolved in gradient TGX Stain-Free Precast Gel (left) and immunostained with anti-Flag Ab (right). Analyzed zone is marked. (**B**) Venn diagram representing proteins identified in three experiments (with triplicate repeats in each) of immunoprecipitation against Flag-tagged human MBP. (**C**) Venn diagram representing unique and reproducible proteins identified in immunoprecipitation against Flag-tagged human MBP compared to negative control.

MBP is characterized by multiple low-affinity semi-specific interactions and therefore combination of fixation of MBP-containing complexes by cross-linking and subsequent highly sensitive LC-MS/MS seems to be the most beneficial methodology. Despite the broad usage of formaldehyde as a crosslinking agent, studying of each particular protein requires optimization of experimental protocol due to the different protein environment, for example in case of cytosolic and membrane proteins [31]. In order to find the optimal conditions of the complex formation and low false-positive rate upon the use of formaldehyde as cross-linking reagent, we varied 2 out of 3 major parameters playing key role in formaldehyde cross-linking: the incubation time and the formaldehyde concentration. We excluded the temperature dependency from the analysis since the minimal effect of room temperature on the crosslinking efficiency was previously shown [32], and the use of other temperature ranges would introduce additional variability due to the difficulty of accurately following the crosslinking protocol. We analyzed the cross-linking efficiency in the range of formaldehyde concentrations from 0.4 to 1% and incubation time from 2 to 10 min and found that the optimal combination of parameters was 0.6% formaldehyde and 10 min of incubation time (data not shown). Upon these parameters, the size of the pool of formed complexes was in the range approximately from 50 to beyond 116 kDa (Figure 2A). It should be noted that under our conditions, the different incubation time in the range of 4–10 min did not result in significant changes in cross-linking. Thus, we chose the interval of 10 min, since it allowed us to minimize timing errors.

A total of three separate cross-linking-IP experiments were carried out with triplicate repeats in each to identify a pull of potential MBP interactors. As a negative control for the IP we used the same lysates and IP procedures, except protein A/G agarose beads (Thermo Fisher Scientific, Waltham, MA, USA) in the control IPs instead of anti-Flag agarose used in the MBP IPs. Final protocol used in the present study reads as follows. HEK293T cells overexpressing Flag-tagged human MBP were exposed to 0.6% formaldehyde for 10 min at room temperature (approximate 23 °C). The formaldehyde-treated cells were washed and lysed in the mild conditions on ice. To remove the lipids and DNA from obtained complexes the lysates were subjected to ultrasound and DNAse treatment. Protein complexes containing MBP were immunoprecipitated from the lysates and the immunoprecipitated complexes was resolved by gradient SDS-PAGE. The protein bands were stained by Coomassie Blue. The gel strips were exized and subjected to mass spectrometry-based proteomics analysis. Cross-linked complexes in a gradient gel gave an about even coloration in the 50-beyond 116 kDa range with several predominant bands (Figure 2A). Therefore, for MS analysis we excised a strip 1 mm wide from the middle of each lane starting from about 30 kDa and up to the top end of the separating gel. The strips were cut in 1 × 1 mm pieces and subjected to the in-gel-trypsinolysis procedure and subsequent MS analysis. Bioinformatic analysis of the obtained proteomic data is presented in Supplementary Table S1. Venn diagram in Figure 2B represents the distribution of the detected proteins between different experiments. The proteins, which were observed in at least two from three MBP IP experiments, and which were either not detected in three control experiments or whose Peptide-Spectrum Matches in MBP experiments were 3.5 or more times over those in control experiments, were considered as possible components of MBP-interacting network.

The detected MBP interactome included 67 different proteins that were identified by Mascot and/or X! Tandem with false discovery rate (FDR) for peptide-spectrum matches less than 0.01 determined by searching a reverse database (Figure 2C).

**Figure 3:**
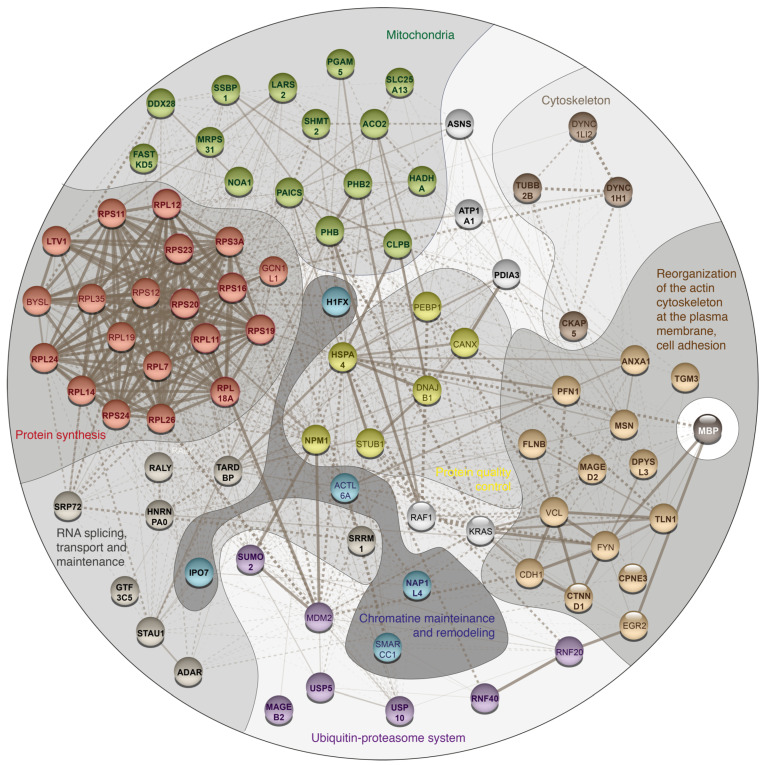
Atlas of the MBP intracellular partners visualized using String database [38,39]. Proteins identified in the current study are shown in bold.

The analysis of identified proteins using String database showed that MBP interacts with eight major proteomic groups presented in Table 1 and Figure 3. The largest group of identified proteins belongs to cellular proteins involved in the protein translation machinery, as well as in the spatial and temporal regulation of translation (Table 1 and Figure 3, red code). As early as 1982, the hypothesis of spatiotemporal regulation of its expression was proposed for the MBP [33]. Subsequently, it was shown that MBP mRNA is transported along microtubules as part of large ribonucleoprotein complexes (granules) to the distal parts of oligodendrocyte processes [34,35]. The local translation of the MBP is initiated by corresponding signaling pathways, triggered by the interaction of the axon with the oligodendrocyte, providing myelination at the contact site [36,37]. The synthesis of MBP is a complex process, which involves not only basic translation proteins, but also proteins that ensure the formation of granules around mRNA, translation regulation, and motor functions [37].

As part of MBP interacting network, we identified core proteins of the ribosome, which belong to 40S subunit–RPS3a, RPS11, RPS16, RPS19, RPS20, RPS23, RPS24 and which are parts of the 60S subunit–RPL7, RPL11, RPL12, RPL14, RPL18a, RPL24, RPL26. MBP was shown to bind protein LTV1 homolog (LTV1), which is responsible for the biogenesis of the small subunit of the ribosome and for its export from the nucleus [40].

Interestingly, the S31 protein from the 28S subunit of the mitochondrial ribosome (MRPS31) was also detected in MBP precipitates (Table 1 and Figure 3, green code). Also, among proteins associated with the mitochondrial ribosome, possible DEAD-box RNA helicase Ddx28 was identified, which helicase activity is necessary for the assembly of the large subunit of the mitochondrial ribosome [41,42,43]. Current data suggest that the spatiotemporal expression of MBP is a complex process, and various ATP-dependent RNA helicases are indispensable factors in its regulation. In 2013, Zhan et al. demonstrated the interaction of DEAD-box RNA helicase Ddx54, which is Ddx28 homolog, with MBP mRNA, and the expression of 21 kDa MBP isoform was dependent on the Ddx54 presence in the cells [44]. Hoch-Kraft et al. showed the presence of Ddx5 in MBP mRNA-associated mRNP complexes in oligodendroglial cells. Ddx5 was shown to mediate alternative splicing and post-transcriptional repression of MBP synthesis in pre-myelinating oligodendrocytes [45]. Among sother MBP interactors, associated with mitochondria, Prohibitin 1 (PHB1) and Prohibitin 2 (PHB2) were also found in the MBP cross-linked protein complexes. Prohibitins form circular structures, alternating with each other in the inner mitochondrial membrane [46]. However, it has recently been shown that Prohibitin 2 is localized at the junction of axon and Schwann cell and required for the myelination process [47]. PHB1 appeared to be not critical for the myelination, but its absence caused severe demyelinating peripheral neuropathy [48].

In the pool of MBP partners we identified several proteins involved in RNA processing and maintenance (Table 1 and Figure 3, dark grey code). In particular, the TAR DNA-binding protein 43 (TARDBP) was detected in MBP precipitates. TARDBP is an RNA-binding protein that is involved in various stages of RNA biogenesis and processing [49,50]. It preferentially binds long clusters of UG-rich sequences in vivo [51]. Aggregation of TARDBP in amyotrophic lateral sclerosis (ALS) could lead to the loss of MBP with compensatory response of oligodendrocytes, which indirectly might serve as an additional evidence for the involvement of TARDBP in the transport of MBP granules [52,53]. The TARDBP was shown to have a functional role in axon–glial interactions in the PNS, and loss of its function in Schwann cells led to impaired conduction velocity and motor behavior [54]. In the MBP bound complex we also identified the RNA-editing enzyme ADAR1 (RNA-specific adenosine deaminase [55]). ADAR1 is important for the normal development of Schwann cells [56]. Cytoplasmic transport of MBP mRNA is mainly dependent on RNA-binding proteins called heterogeneous nuclear ribonucleoproteins (hnRNP). In complex with MBP we have identified hnRNP A0, which belongs to the family of hnRNP A/B, recognizing AU-rich elements (ARE) of mRNA. Another member of this family, hnRNP A2 recognizes the A2RE element at the 3′-end of MBP mRNA [57]. The increase of hnRNP A2 protein level in the cytoplasm of oligodendrocytes correlated with the timeline of MBP gene expression induction and rapid MBP accumulation [58]. In addition, the presence of heterogeneous nuclear ribonucleoproteins hnRNP K, E1, F, and CBF-A in the complex with MBP mRNA was previously shown [59,60].

One more representative group of cellular proteins involved in regulation of MBP synthesis contains proteins participating in the processes of the reorganization of the actin cytoskeleton and intercellular adhesion, including those at the site of contact between the oligodendrocyte and axon (Table 1 and Figure 3, light brown code). Intercellular interaction proteins trigger intracellular signaling pathways that provide derepression of MBP synthesis at the site of contact. In the MBP-interacting protein network, we identified the protein catenin delta 1 (CTNND1), which is necessary for myelinating Schwann cells, cell-cell interactions and the formation of the normal myelin sheath [61]. CTNND1 is involved in the regulation of cadherin-mediated adhesion and dynamic regulation of the actin cytoskeleton by modulating the activity of Rho GTPase [62,63,64]. The mechanism of MBP translation derepression at the local contact site of axon and oligodendrocyte is mediated by integrin-dependent activation of Src-family kinase Fyn. Previously Fyn was found in immunocomplexes with MBP in oligodendrocytes [65]. CNS myelination of *fyn*^−/−^ null mutant mice was severely disrupted, yet myelin content in null mutants lacking the Fyn-related kinases Src, Yes, or Lyn was unaffected, and the myelin deficit was a result of inhibition of Fyn tyrosine kinase activity [66,67]. Fyn kinase also mediates the promyelinating influence of BDNF. In this process, the phosphorylation of Fyn is provided by oligodendroglial TrkB receptors, activated by BDNF [68]. Fyn expression was upregulated upon induced differentiation of the CG4 oligodendrocyte cell line, consistent with the observation that Fyn was enriched in the compact myelin and gradually declined with age [69]. The hnRNP A2 was identified as one of Fyn substrates in oligodendrocytes. Its activation in oligodendrocytes is controlled by a signaling cascade at the point of contact between the axon and the glial cell [70] providing the link between Fyn and activation of MBP synthesis.

The cytoskeleton adaptor protein Talin 1 (TLN1), which we identified in complex with MBP, plays a central role in the functioning of integrins. It is required for the formation of a mechanical bond between integrins and the actin cytoskeleton, and is a necessary regulator of integrin activation and the subsequent signaling cascade [71,72,73,74,75]. Among proteins that bind the membrane and actin cytoskeleton, we identified Filamin B, which is potentially involved in the traffic of MBP mRNA-containing granules [76,77]. Another protein observed in the pool of MBP interacting proteins is Moesin (MSN). Moesin and its analogs ezrin and radixin belong to a family of closely related ERM proteins and bind actin filaments and the plasma membrane. They form membrane-cytoskeletal bonds and serve as a linkers between the plasma membrane and actin cytoskeleton [78]. These proteins play a key role in the adhesion, migration and organization of the cell surface structures. Expression of ezrin, moezin paralog, is specific for Schwann cells where it is involved in maintaining of the integrity of the myelin sheath [79,80]. In the pool of MBP interacting proteins, Annexin A1 (ANXA1) and Copine III (CPNE3) proteins were also found, probably because they bind to phospholipids like MBP. Annexin A1 (lipocortin 1) is a calcium and phospholipid-binding protein with anti-inflammatory properties [81], which also regulates the cytoskeleton via actin and profilin [82]. Notably, Profilin-1 (PFN1) was also found in the MBP-interacting network. With respect to neurodegenerative diseases, ANXA1 has been shown to accumulate in plaques associated with multiple sclerosis [83]. Additionally, ANXA1 mitigates the course of the experimental autoimmune encephalomyelitis [84]. The content of ANXA1 in cerebral vascular endothelial cells decreases when the cells are exposed to the serum of patients with active multiple sclerosis [85]. In a complex with MBP we identified representatives of another group of cytoskeleton and intracellular traffic proteins (Table 1 and Figure 3, deep brown code), namely Cytoskeleton-associated protein 5, Cytoplasmic dynein 1 heavy chain 1 and Tubulin beta chain. Previously, it was shown that MBP co-precipitates with microtubules from the human brain [86] and interacts with β-tubulin [65].

In the complex with MBP, two proteins of the family of melanoma associated genes B2 and D2, MAGEB2 and MAGED2 were identified (Table 1 and Figure 3, violet/light brown code). This family includes several dozen genes that share a common MAGE homology domain (MHD). MAGED1 has been shown to be associated with p75 neurotrophin receptor (p75NTR), functioning as an adaptor that mediates multiple signaling pathways [87]. The p75NTR is a positive regulator of myelination processes [88]. Recent biochemical and biophysical studies have shown that proteins from the MAGE family are associated with E3 RING ubiquitin ligases to form MAGE-RING ligases (MRLs) and act as regulators of ubiquitination by modulating ligase activity, substrate specificity, and intracellular localization [89]. In association with MBP we also identified the RNF40–the RNF20/40 E3 RING ubiquitin ligase subunit (Table 1 and Figure 3, violet code). The E3 ligase containing RNF40 monoubiquinates histone H2B where it is recruited by the transcription factor Egr2, which is the central transcriptional regulator of peripheral myelination [90]. Xie et al. demonstrated that H2B monoubiquitination by RNF20/40 E3 RING ubiquitin ligase is crucial for the induced pluripotent stem cells (iPSCs) formation in somatic cell reprogramming. Deletion of Rnf40 affected cell lineage-specific gene silencing and pluripotency gene activation [91]. In consistency with this, H2B ubiquitin ligase RNF40 is required for the induction of differentiation markers and transcriptional reprogramming of human mesenchymal stem cells (hMSCs) [92]. Among other members of ubiquitin-proteasome system, interacting with MBP, we identified ubiquitin carboxyl-terminal hydrolases 5 and 10 (USP5 and USP10), components of the SMC5-SMC6 complex, as well as Small Ubiquitin-like Modifiers 2 and 3 (SUMO2/3).

Previously it was shown that all main isoforms of MBP are localized in the nucleus, and its transport to the nucleus is actively regulated [93]. Among the proteins found in complex with MBP there are nuclear proteins responsible for chromatin remodeling and maintenance of DNA structure (Table 1 and Figure 3, cyan code). Histone H1.X, found in a complex with MBP, belongs to H1 histone type. Histone H1.X stabilizes higher order chromatin folding through stabilization of chromatin compaction [94]. H1 linker histones bind to the proteins of the NAP (Nucleosome assembly protein) family, which includes the identified Nucleosome assembly protein 1-like 4 1 (NAP1L4) [95]. Importin-7 (IPO7), which is possibly associated with the import of MBP into the nucleus, was also identified in precipitated MBP complexes.

All MBP-associated protein groups are interconnected with each other through members of protein quality control system, namely the nucleophosmin 1 (NPM1) and Heat shock 70 kDa protein 4 (HSPA4) (Table 1 and Figure 3, yellow code). HSPs are a key component of the chaperone-assisted refolding, which provides protein quality control by specific recognition of the misfolded proteins [96]. Optimal expression of myelin basic protein during differentiation of the oligodendrocytes requires the constitutive heat shock protein-70 [97], probably due to the intrinsically-disordered nature of the MBP. A multifunctional nucleolar organizer-NPM1 is homopentamer with globular domains connected by long, intrinsically disordered linkers [98]. NPM1 has the crucial functions in cell growth and homeostasis, including regulation of ribosome biogenesis and stress response [99]. Such multiple activities rely on its ability to interact with hundreds of proteins, including multivalent interactions with proteins containing arginine-rich linear motifs (R-motifs), nucleic acids and ribosomal RNA. NPM1 modulates multiple mechanisms of liquid-liquid phase separation (LLPS) phenomena, which, as becoming well accepted, underlie a key role in nucleolar organization [100]. LLPS occurs in the intracellular membraneless organelles, which represent a protein/nuclear acid-rich phase coexisting with a protein-poor bulk phase. Intrinsically disordered proteins (such as MBP) are easily undergo LLPS [101]. Moreover, LLPS-mediated nuclear quality control mechanism is perturbed during neurodegeneration [102].

## 4. Conclusions

Elucidation of the mechanisms that guide myelin membrane biogenesis may result not only in deeper understanding of the pathogenesis of the autoimmune neurodegeneration, but also in novel therapeutic approaches. Here using combination of formaldehyde-induced cross-linking followed by immunoprecipitation and liquid chromatography-tandem mass spectrometry (LC-MS/MS) we showed that MBP interacts not only with structural membrane-associated and cytoskeletal proteins, but also with proteins related to protein expression starting from transcription-translation machinery and continuing with mitochondrial proteins, transport and protein folding systems. Concluding, our data suggest that MBP may have not only structural but also regulatory role in myelinization process, which is significantly more proactive that was believed previously.

## Figures and Tables

**Table 1:** List of potential MBP interacting proteins. PSMs—Peptide-Spectrum Matches.

Protein Name	Gene Name	PSMs for MBP Experiment			PSMs for Control Experiment		
Protein synthesis							
40S ribosomal protein S16	RPS16	0	1	2	0	0	0
40S ribosomal protein S19	RPS19	0	1	1	0	0	0
40S ribosomal protein S20	RPS20	0	2	4	0	0	0
40S ribosomal protein S23	RPS23	3	0	4	0	0	0
60S ribosomal protein L11	RPL11	0	1	3	0	0	0
60S ribosomal protein L12	RPL12	0	1	1	0	0	0
60S ribosomal protein L18a	RPL18A	0	3	8	0	0	0
Protein LTV1 homolog	LTV1	0	2	1	0	0	0
Ribosomal protein L7, isoform CRA_a	RPL7	0	1	4	0	0	0
40S ribosomal protein S24	RPS24	0	2	3	0	0	1
40S ribosomal protein S11	RPS11	0	1	8	0	0	2
60S ribosomal protein L26	RPL26	0	3	6	0	0	2
40S ribosomal protein S3a	RPS3A	3	2	12	0	0	4
60S ribosomal protein L24	RPL24	0	2	2	0	0	1
60S ribosomal protein L14	RPL14	0	2	5	0	0	2
Mitochondria							
28S ribosomal protein S31, mitochondrial	MRPS31	0	2	2	0	0	0
Aconitate hydratase, mitochondrial	ACO2	1	0	2	0	0	0
cDNA FLJ46863 fis, clone UTERU3011558	NOA1	1	0	2	0	0	0
FAST kinase domain-containing protein 5, mitochondrial	FASTKD5	0	2	1	0	0	0
Isoform 2 of Caseinolytic peptidase B protein homolog	CLPB	0	2	8	0	0	0
Prohibitin (Fragment)	PHB1	2	2	3	0	0	0
Prohibitin-2	PHB2	5	2	6	0	0	0
Single-stranded DNA-binding protein, mitochondrial (Fragment)	SSBP1	1	0	2	0	0	0
Solute carrier family 25, member 13 (Citrin) variant (Fragment)	SLC25A13	0	1	3	0	0	0
Probable ATP-dependent RNA helicase DDX28	DDX28	0	2	6	0	0	1
Enoyl-CoA hydratase	HADHA	3	1	7	0	0	2
Probable leucine--tRNA ligase, mitochondrial	LARS2	0	2	3	0	0	1
AIR carboxylase (Fragment)	PAICS	2	0	6	0	0	2
Serine hydroxymethyltransferase	SHMT2	5	3	17	0	0	7
Serine/threonine-protein phosphatase PGAM5, mitochondrial	PGAM5	1	0	6	0	0	2
mRNA splicing, transport and maintenance							
cDNA FLJ77421, highly similar to Homo sapiens autoantigen p542 mRNA	RALY	2	1	0	0	0	0
Isoform 2 of General transcription factor 3C polypeptide 5	GTF3C5	0	2	1	0	0	0
Serine/arginine repetitive matrix 1 isoform 2 (Fragment)	SRRM1	0	4	1	0	0	0
Heterogeneous nuclear ribonucleoprotein A0	HNRNPA0	2	2	1	0	0	1
Signal recognition particle subunit SRP72	SRP72	1	0	4	0	0	1
cDNA FLJ75871, highly similar to Homo sapiens staufen, RNA binding protein (STAU), transcript variant T3, mRNA	STAU1	0	5	8	0	0	3
RNA-specific adenosine deaminase	ADAR	3	3	1	0	2	0
TAR DNA-binding protein 43	TARDBP	4	1	2	0	0	2
Reorganization of the cytoskeleton and intercellular adhesion							
Annexin A1	ANXA1	4	3	2	0	0	0
Catenin delta-1	CTNND1	0	1	5	0	0	0
Copine III, isoform CRA_a	CPNE3	0	2	2	0	0	0
Filamin B, beta (Actin binding protein 278), isoform CRA_a	FLNB	2	1	4	0	0	0
Moesin	MSN	2	0	8	0	1	1
cDNA FLJ56823, highly similar to Protein-glutamine gamma-glutamyltransferase E	TGM3	1	1	2	0	1	0
Profilin-1	PFN1	2	1	1	0	0	1
Talin-1	TLN1	3	4	16	0	1	5
Testicular secretory protein Li 7	DPYSL3	0	2	5	0	1	1
Cytoskeleton and intracellular traffic							
Cytoskeleton-associated protein 5	CKAP5	1	0	8	0	0	1
Cytoplasmic dynein 1 heavy chain 1	DYNC1H1	3	0	1	0	0	1
Tubulin beta chain	TUBB2B	0	2	5	0	0	2
Ubiquitin-proteasome system related							
cDNA, FLJ93871, highly similar to Homo sapiens melanoma antigen, family B, 2 (MAGEB2), mRNA	MAGEB2	0	1	3	0	0	0
E3 ubiquitin protein ligase	RNF40	0	2	1	0	0	0
Small ubiquitin-related modifier	SUMO2	0	2	2	0	0	0
Ubiquitin carboxyl-terminal hydrolase	USP10	0	2	1	0	0	0
Melanoma antigen family D, 2, isoform CRA_a	MAGED2	0	2	11	0	0	1
Ubiquitin carboxyl-terminal hydrolase	USP5	1	1	5	0	0	1
Quality control proteins							
Heat shock 70 kDa protein 4	HSPA4	2	0	1	0	0	0
Nucleophosmin (Fragment)	NPM1	11	3	6	0	0	3
Chromatin mainteinance and remodeling							
Nucleosome assembly protein 1-like 4, isoform CRA_b	NAP1L4	1	0	3	0	0	0
Histone H1.10	H1-10	2	1	0	0	0	0
Importin-7	IPO7	0	2	7	0	0	1
Unassigned to the specific group proteins							
Asparagine synthetase [glutamine-hydrolyzing]	ASNS	0	3	5	0	0	1
Protein disulfide-isomerase A3 (Fragment)	PDIA3	4	0	1	0	0	1
Sodium/potassium-transporting ATPase subunit alpha (Fragment)	ATP1A1	0	1	3	0	0	1
BAF53A protein	BAF53A	2	0	1	0	0	0
Peroxisome proliferator activated receptor interacting complex protein	PRIC295	1	0	9	0	0	1

## Data Availability

Publicly available datasets were analyzed in this study. This data can be found here: https://www.ebi.ac.uk/pride/archive/projects/PXD028685, accessed on 1 February 2020.

## Supplementary Materials

The following are available online at https://www.mdpi.com/article/10.3390/biom11111628/s1, Table S1. Summary table of identified proteins. Summary table of identified proteins. Sheet 1—Description of samples, total number of identified proteins and spectra for each analyzed sample. Sheet 2—List of identified peptide sequences. Sheet 3—List of identified proteins.

## References

[1] Benjamins J.A., Morell P. (1978). Proteins of Myelin and Their Metabolism. Neurochem. Res..

[2] Garbay B., Fournier M., Sallafranque M.L., Muller S., Boiron F., Heape A., Cassagne C., Bonnet J. (1988). Po, MBP, Histone, and DNA Levels in Sciatic Nerve. Neurochem. Pathol..

[3] Krigbaum W.R., Hsu T.S. (1975). Molecular Conformation of Bovine A1 Basic Protein, a Coiling Macromolecule in Aqueous Solution. Biochemistry.

[4] Polverini E., Fasano A., Zito F., Riccio P., Cavatorta P. (1999). Conformation of Bovine Myelin Basic Protein Purified with Bound Lipids. Eur. Biophys. J. EBJ.

[5] Harauz G., Ishiyama N., Hill C.M.D., Bates I.R., Libich D.S., Farès C. (2004). Myelin Basic Protein-Diverse Conformational States of an Intrinsically Unstructured Protein and Its Roles in Myelin Assembly and Multiple Sclerosis. Micron.

[6] Harauz G., Libich D. (2009). The Classic Basic Protein of Myelin—Conserved Structural Motifs and the Dynamic Molecular Barcode Involved in Membrane Adhesion and Protein-Protein Interactions. Curr. Protein Pept. Sci..

[7] Aggarwal S., Snaidero N., Pähler G., Frey S., Sánchez P., Zweckstetter M., Janshoff A., Schneider A., Weil M.-T., Schaap I.A.T. (2013). Myelin Membrane Assembly Is Driven by a Phase Transition of Myelin Basic Proteins Into a Cohesive Protein Meshwork. PLoS Biol..

[8] Boggs J.M. (2006). Myelin Basic Protein: A Multifunctional Protein. Cell. Mol. Life Sci. CMLS.

[9] Boggs J.M., Yip P.M., Rangaraj G., Jo E. (1997). Effect of Posttranslational Modifications to Myelin Basic Protein on Its Ability to Aggregate Acidic Lipid Vesicles. Biochemistry.

[10] Bates I.R., Boggs J.M., Feix J.B., Harauz G. (2003). Membrane-Anchoring and Charge Effects in the Interaction of Myelin Basic Protein with Lipid Bilayers Studied by Site-Directed Spin Labeling. J. Biol. Chem..

[11] Hill C.M.D., Harauz G. (2005). Charge Effects Modulate Actin Assembly by Classic Myelin Basic Protein Isoforms. Biochem. Biophys. Res. Commun..

[12] Homchaudhuri L., Polverini E., Gao W., Harauz G., Boggs J.M. (2009). Influence of Membrane Surface Charge and Post-Translational Modifications to Myelin Basic Protein on Its Ability to Tether the Fyn-SH3 Domain to a Membrane in Vitro. Biochemistry.

[13] Belogurov A., Kudriaeva A., Kuzina E., Smirnov I., Bobik T., Ponomarenko N., Kravtsova-Ivantsiv Y., Ciechanover A., Gabibov A. (2014). Multiple Sclerosis Autoantigen Myelin Basic Protein Escapes Control by Ubiquitination during Proteasomal Degradation. J. Biol. Chem..

[14] Kudriaeva A., Kuzina E.S., Zubenko O., Smirnov I.V., Belogurov A. (2019). Charge-Mediated Proteasome Targeting. FASEB J..

[15] Lutton J.D., Winston R., Rodman T.C. (2004). Multiple Sclerosis: Etiological Mechanisms and Future Directions. Exp. Biol. Med..

[16] Sospedra M., Martin R. (2005). Immunology of Multiple Sclerosis. Annu. Rev. Immunol..

[17] Belogurov A.A., Kurkova I.N., Friboulet A., Thomas D., Misikov V.K., Zakharova M.Y., Suchkov S.V., Kotov S.V., Alehin A.I., Avalle B. (2008). Recognition and Degradation of Myelin Basic Protein Peptides by Serum Autoantibodies: Novel Biomarker for Multiple Sclerosis. J. Immunol. 1950.

[18] Ponomarenko N.A., Durova O.M., Vorobiev I.I., Belogurov A.A., Kurkova I.N., Petrenko A.G., Telegin G.B., Suchkov S.V., Kiselev S.L., Lagarkova M.A. (2006). Autoantibodies to Myelin Basic Protein Catalyze Site-Specific Degradation of Their Antigen. Proc. Natl. Acad. Sci. USA.

[19] Ponomarenko N.A., Durova O.M., Vorobiev I.I., Belogurov A.A., Telegin G.B., Suchkov S.V., Misikov V.K., Morse H.C., Gabibov A.G. (2006). Catalytic Activity of Autoantibodies toward Myelin Basic Protein Correlates with the Scores on the Multiple Sclerosis Expanded Disability Status Scale. Immunol. Lett..

[20] Kuzina E.S., Chernolovskaya E.L., Kudriaeva A.A., Zenkova M.A., Knorre V.D., Surina E.A., Ponomarenko N.A., Bobik T.V., Smirnov I.V., Bacheva A.V. (2013). Immunoproteasome Enhances Intracellular Proteolysis of Myelin Basic Protein. Dokl. Biochem. Biophys..

[21] Kim J.K., Mastronardi F.G., Wood D.D., Lubman D.M., Zand R., Moscarello M.A. (2003). Multiple Sclerosis: An Important Role for Post-Translational Modifications of Myelin Basic Protein in Pathogenesis. Mol. Cell. Proteom..

[22] Kuzina E.S., Kudriaeva A.A., Glagoleva I.S., Knorre V.D., Gabibov A.G., Belogurov A.A. (2016). Deimination of the Myelin Basic Protein Decelerates Its Proteasome-Mediated Metabolism. Dokl. Biochem. Biophys..

[23] Harauz G., Musse A.A. (2007). A Tale of Two Citrullines—Structural and Functional Aspects of Myelin Basic Protein Deimination in Health and Disease. Neurochem. Res..

[24] Wang C., Neugebauer U., Bürck J., Myllykoski M., Baumgärtel P., Popp J., Kursula P. (2011). Charge Isomers of Myelin Basic Protein: Structure and Interactions with Membranes, Nucleotide Analogues, and Calmodulin. PLoS ONE.

[25] Kuzina E., Kudriaeva A., Smirnov I., Dubina M.V., Gabibov A., Belogurov A. (2014). Glatiramer Acetate and Nanny Proteins Restrict Access of the Multiple Sclerosis Autoantigen Myelin Basic Protein to the 26S Proteasome. BioMed Res. Int..

[26] Belogurov A., Kuzina E., Kudriaeva A., Kononikhin A., Kovalchuk S., Surina Y., Smirnov I., Lomakin Y., Bacheva A., Stepanov A. (2015). Ubiquitin-Independent Proteosomal Degradation of Myelin Basic Protein Contributes to Development of Neurodegenerative Autoimmunity. FASEB J..

[27] Shevchenko A., Tomas H., Havli J., Olsen J.V., Mann M. (2006). In-Gel Digestion for Mass Spectrometric Characterization of Proteins and Proteomes. Nat. Protoc..

[28] Kovalchuk S.I., Jensen O.N., Rogowska-Wrzesinska A. (2019). FlashPack: Fast and Simple Preparation of Ultrahigh-Performance Capillary Columns for LC-MS. Mol. Cell. Proteom..

[29] Perez-Riverol Y., Csordas A., Bai J., Bernal-Llinares M., Hewapathirana S., Kundu D.J., Inuganti A., Griss J., Mayer G., Eisenacher M. (2019). The PRIDE Database and Related Tools and Resources in 2019: Improving Support for Quantification Data. Nucleic Acids Res..

[30] Deutsch E.W., Bandeira N., Sharma V., Perez-Riverol Y., Carver J.J., Kundu D.J., García-Seisdedos D., Jarnuczak A.F., Hewapathirana S., Pullman B.S. (2020). The ProteomeXchange Consortium in 2020: Enabling “big Data” Approaches in Proteomics. Nucleic Acids Res..

[31] Sutherland B.W., Toews J., Kast J. (2008). Utility of Formaldehyde Cross-Linking and Mass Spectrometry in the Study of Protein–Protein Interactions. J. Mass Spectrom..

[32] Klockenbusch C., Kast J. (2010). Optimization of Formaldehyde Cross-Linking for Protein Interaction Analysis of Non-Tagged Integrin β 1. J. Biomed. Biotechnol..

[33] Colman D.R., Kreibich G., Frey A.B., Sabatini D.D. (1982). Synthesis and Incorporation of Myelin Polypeptides into CNS Myelin. J. Cell Biol..

[34] Ainger K., Avossa D., Morgan F., Hill S.J., Barry C., Barbarese E., Carson J.H. (1993). Transport and Localization of Exogenous Myelin Basic Protein MRNA Microinjected into Oligodendrocytes. J. Cell Biol..

[35] Carson J.H., Worboys K., Ainger K., Barbarese E. (1997). Translocation of Myelin Basic Protein MRNA in Oligodendrocytes Requires Microtubules and Kinesin. Cell Motil. Cytoskelet..

[36] Klein C., Kramer E.-M., Cardine A.-M., Schraven B., Brandt R., Trotter J. (2002). Process Outgrowth of Oligodendrocytes Is Promoted by Interaction of Fyn Kinase with the Cytoskeletal Protein Tau. J. Neurosci..

[37] Müller C., Bauer N.M., Schäfer I., White R. (2013). Making Myelin Basic Protein-from MRNA Transport to Localized Translation. Front. Cell. Neurosci..

[38] Szklarczyk D., Gable A.L., Nastou K.C., Lyon D., Kirsch R., Pyysalo S., Doncheva N.T., Legeay M., Fang T., Bork P. (2021). The STRING Database in 2021: Customizable Protein-Protein Networks, and Functional Characterization of User-Uploaded Gene/Measurement Sets. Nucleic Acids Res..

[39] Szklarczyk D., Gable A.L., Lyon D., Junge A., Wyder S., Huerta-Cepas J., Simonovic M., Doncheva N.T., Morris J.H., Bork P. (2019). STRING V11: Protein-Protein Association Networks with Increased Coverage, Supporting Functional Discovery in Genome-Wide Experimental Datasets. Nucleic Acids Res..

[40] Granneman S., Petfalski E., Swiatkowska A., Tollervey D. (2010). Cracking Pre-40S Ribosomal Subunit Structure by Systematic Analyses of RNA-Protein Cross-Linking. EMBO J..

[41] Valgardsdottir R., Brede G., Eide L.G., Frengen E., Prydz H. (2001). Cloning and Characterization of MDDX28, a Putative Dead-Box Helicase with Mitochondrial and Nuclear Localization. J. Biol. Chem..

[42] Tu Y.-T., Barrientos A. (2015). The Human Mitochondrial DEAD-Box Protein DDX28 Resides in RNA Granules and Functions in Mitoribosome Assembly. Cell Rep..

[43] Antonicka H., Shoubridge E.A. (2015). Mitochondrial RNA Granules Are Centers for Posttranscriptional RNA Processing and Ribosome Biogenesis. Cell Rep..

[44] Zhan R., Yamamoto M., Ueki T., Yoshioka N., Tanaka K., Morisaki H., Seiwa C., Yamamoto Y., Kawano H., Tsuruo Y. (2013). A DEAD-Box RNA Helicase Ddx54 Protein in Oligodendrocytes Is Indispensable for Myelination in the Central Nervous System. J. Neurosci. Res..

[45] Hoch-Kraft P., White R., Tenzer S., Krämer-Albers E.-M., Trotter J., Gonsior C. (2018). Dual Role of the RNA Helicase DDX5 in Post-Transcriptional Regulation of Myelin Basic Protein in Oligodendrocytes. J. Cell Sci..

[46] Tatsuta T., Model K., Langer T. (2005). Formation of Membrane-Bound Ring Complexes by Prohibitins in Mitochondria. Mol. Biol. Cell.

[47] Poitelon Y., Bogni S., Matafora V., Della-Flora Nunes G., Hurley E., Ghidinelli M., Katzenellenbogen B.S., Taveggia C., Silvestri N., Bachi A. (2015). Spatial Mapping of Juxtacrine Axo-Glial Interactions Identifies Novel Molecules in Peripheral Myelination. Nat. Commun..

[48] Della-Flora Nunes G., Wilson E.R., Marziali L.N., Hurley E., Silvestri N., He B., O’Malley B.W., Beirowski B., Poitelon Y., Wrabetz L. (2021). Prohibitin 1 Is Essential to Preserve Mitochondria and Myelin Integrity in Schwann Cells. Nat. Commun..

[49] Bhardwaj A., Myers M.P., Buratti E., Baralle F.E. (2013). Characterizing TDP-43 Interaction with Its RNA Targets. Nucleic Acids Res..

[50] Tollervey J.R., Curk T., Rogelj B., Briese M., Cereda M., Kayikci M., König J., Hortobágyi T., Nishimura A.L., Zupunski V. (2011). Characterizing the RNA Targets and Position-Dependent Splicing Regulation by TDP-43. Nat. Neurosci..

[51] Wang A., Conicella A.E., Schmidt H.B., Martin E.W., Rhoads S.N., Reeb A.N., Nourse A., Ramirez Montero D., Ryan V.H., Rohatgi R. (2018). A Single N-Terminal Phosphomimic Disrupts TDP-43 Polymerization, Phase Separation, and RNA Splicing. EMBO J..

[52] Rohan Z., Matej R., Rusina R., Kovacs G.G. (2014). Oligodendroglial Response in the Spinal Cord in TDP-43 Proteinopathy with Motor Neuron Involvement. Neurodegener. Dis..

[53] Masaki K., Sonobe Y., Ghadge G., Pytel P., Lépine P., Pernin F., Cui Q.-L., Antel J.P., Zandee S., Prat A. (2020). RNA-Binding Protein Altered Expression and Mislocalization in MS. Neurol. Neuroimmunol. Neuroinflamm..

[54] Chang K.-J., Agrawal I., Vainshtein A., Ho W.Y., Xin W., Tucker-Kellogg G., Susuki K., Peles E., Ling S.-C., Chan J.R. (2021). TDP-43 Maximizes Nerve Conduction Velocity by Repressing a Cryptic Exon for Paranodal Junction Assembly in Schwann Cells. eLife.

[55] Liu Y., Herbert A., Rich A., Samuel C.E. (1998). Double-Stranded RNA-Specific Adenosine Deaminase: Nucleic Acid Binding Properties. Methods.

[56] Gacem N., Kavo A., Zerad L., Richard L., Mathis S., Kapur R.P., Parisot M., Amiel J., Dufour S., de la Grange P. (2020). ADAR1 Mediated Regulation of Neural Crest Derived Melanocytes and Schwann Cell Development. Nat. Commun..

[57] Hoek K.S., Kidd G.J., Carson J.H., Smith R. (1998). HnRNP A2 Selectively Binds the Cytoplasmic Transport Sequence of Myelin Basic Protein MRNA. Biochemistry.

[58] Maggipinto M., Rabiner C., Kidd G.J., Hawkins A.J., Smith R., Barbarese E. (2004). Increased Expression of the MBP MRNA Binding Protein HnRNP A2 during Oligodendrocyte Differentiation. J. Neurosci. Res..

[59] Raju C.S., Göritz C., Nord Y., Hermanson O., López-Iglesias C., Visa N., Castelo-Branco G., Percipalle P. (2008). In Cultured Oligodendrocytes the A/B-Type HnRNP CBF-A Accompanies MBP MRNA Bound to MRNA Trafficking Sequences. Mol. Biol. Cell.

[60] White R., Gonsior C., Bauer N.M., Krämer-Albers E.-M., Luhmann H.J., Trotter J. (2012). Heterogeneous Nuclear Ribonucleoprotein (HnRNP) F Is a Novel Component of Oligodendroglial RNA Transport Granules Contributing to Regulation of Myelin Basic Protein (MBP) Synthesis. J. Biol. Chem..

[61] Perrin-Tricaud C., Rutishauser U., Tricaud N. (2007). P120 Catenin Is Required for Thickening of Schwann Cell Myelin. Mol. Cell. Neurosci..

[62] Anastasiadis P.Z., Moon S.Y., Thoreson M.A., Mariner D.J., Crawford H.C., Zheng Y., Reynolds A.B. (2000). Inhibition of RhoA by P120 Catenin. Nat. Cell Biol..

[63] Grosheva I., Shtutman M., Elbaum M., Bershadsky A.D. (2001). P120 Catenin Affects Cell Motility via Modulation of Activity of Rho-Family GTPases: A Link between Cell-Cell Contact Formation and Regulation of Cell Locomotion. J. Cell Sci..

[64] Noren N.K., Liu B.P., Burridge K., Kreft B. (2000). P120 Catenin Regulates the Actin Cytoskeleton via Rho Family GTPases. J. Cell Biol..

[65] Boggs J.M., Homchaudhuri L., Ranagaraj G., Liu Y., Smith G.S., Harauz G. (2014). Interaction of Myelin Basic Protein with Cytoskeletal and Signaling Proteins in Cultured Primary Oligodendrocytes and N19 Oligodendroglial Cells. BMC Res. Notes.

[66] Sperber B.R., Boyle-Walsh É.A., Engleka M.J., Gadue P., Peterson A.C., Stein P.L., Scherer S.S., McMorris F.A. (2001). A Unique Role for Fyn in CNS Myelination. J. Neurosci..

[67] Krämer-Albers E.-M., White R. (2011). From Axon–Glial Signalling to Myelination: The Integrating Role of Oligodendroglial Fyn Kinase. Cell. Mol. Life Sci..

[68] Peckham H., Giuffrida L., Wood R., Gonsalvez D., Ferner A., Kilpatrick T.J., Murray S.S., Xiao J. (2016). Fyn Is an Intermediate Kinase That BDNF Utilizes to Promote Oligodendrocyte Myelination. Glia.

[69] Lu Z., Ku L., Chen Y., Feng Y. (2005). Developmental Abnormalities of Myelin Basic Protein Expression in Fyn Knock-out Brain Reveal a Role of Fyn in Posttranscriptional Regulation. J. Biol. Chem..

[70] White R., Gonsior C., Krämer-Albers E.-M., Stöhr N., Hüttelmaier S., Trotter J. (2008). Activation of Oligodendroglial Fyn Kinase Enhances Translation of MRNAs Transported in HnRNP A2–Dependent RNA Granules. J. Cell Biol..

[71] Bouaouina M., Harburger D.S., Calderwood D.A. (2012). Talin and Signaling through Integrins. Methods Mol. Biol..

[72] Calderwood D.A., Ginsberg M.H. (2003). Talin Forges the Links between Integrins and Actin. Nat. Cell Biol..

[73] Critchley D.R. (2009). Biochemical and Structural Properties of the Integrin-Associated Cytoskeletal Protein Talin. Annu. Rev. Biophys..

[74] Klapholz B., Brown N.H. (2017). Talin—The Master of Integrin Adhesions. J. Cell Sci..

[75] Moser M., Legate K.R., Zent R., Fässler R. (2009). The Tail of Integrins, Talin, and Kindlins. Science.

[76] Lu J., Lian G., Lenkinski R., De Grand A., Vaid R.R., Bryce T., Stasenko M., Boskey A., Walsh C., Sheen V. (2007). Filamin B Mutations Cause Chondrocyte Defects in Skeletal Development. Hum. Mol. Genet..

[77] Zhang W., Han S.W., McKeel D.W., Goate A., Wu J.Y. (1998). Interaction of Presenilins with the Filamin Family of Actin-Binding Proteins. J. Neurosci..

[78] Vaheri A., Carpén O., Heiska L., Helander T.S., Jääskeläinen J., Majander-Nordenswan P., Sainio M., Timonen T., Turunen O. (1997). The Ezrin Protein Family: Membrane-Cytoskeleton Interactions and Disease Associations. Curr. Opin. Cell Biol..

[79] Guo T., Zhang L., Xiao H., Yang Y., Shi Y. (2020). Ezrin Interacts with L-Periaxin by the “Head to Head and Tail to Tail” Mode and Influences the Location of L-Periaxin in Schwann Cell RSC96. Biochim. Biophys. Acta.

[80] Melendez-Vasquez C.V., Rios J.C., Zanazzi G., Lambert S., Bretscher A., Salzer J.L. (2001). Nodes of Ranvier Form in Association with Ezrin-Radixin-Moesin (ERM)-Positive Schwann Cell Processes. Proc. Natl. Acad. Sci. USA.

[81] Perretti M., D’Acquisto F. (2009). Annexin A1 and Glucocorticoids as Effectors of the Resolution of Inflammation. Nat. Rev. Immunol..

[82] Alvarez-Martinez M.T., Porte F., Liautard J.P., Sri Widada J. (1997). Effects of Profilin-Annexin I Association on Some Properties of Both Profilin and Annexin I: Modification of the Inhibitory Activity of Profilin on Actin Polymerization and Inhibition of the Self-Association of Annexin I and Its Interactions with Liposomes. Biochim. Biophys. Acta.

[83] Probst-Cousin S., Kowolik D., Kuchelmeister K., Kayser C., Neundörfer B., Heuss D. (2002). Expression of Annexin-1 in Multiple Sclerosis Plaques. Neuropathol. Appl. Neurobiol..

[84] Huitinga I., Bauer J., Strijbos P.J., Rothwell N.J., Dijkstra C.D., Tilders F.J. (1998). Effect of Annexin-1 on Experimental Autoimmune Encephalomyelitis (EAE) in the Rat. Clin. Exp. Immunol..

[85] Alexander J.S., Minagar A., Harper M., Robinson-Jackson S., Jennings M., Smith S.J. (2007). Proteomic Analysis of Human Cerebral Endothelial Cells Activated by Multiple Sclerosis Serum and IFNβ-1b. J. Mol. Neurosci..

[86] Taketomi M., Kinoshita N., Kimura K., Kitada M., Noda T., Asou H., Nakamura T., Ide C. (2002). Nogo-A Expression in Mature Oligodendrocytes of Rat Spinal Cord in Association with Specific Molecules. Neurosci. Lett..

[87] Salehi A.H., Roux P.P., Kubu C.J., Zeindler C., Bhakar A., Tannis L.-L., Verdi J.M., Barker P.A. (2000). NRAGE, A Novel MAGE Protein, Interacts with the P75 Neurotrophin Receptor and Facilitates Nerve Growth Factor–Dependent Apoptosis. Neuron.

[88] Cosgaya J.M. (2002). The Neurotrophin Receptor P75NTR as a Positive Modulator of Myelination. Science.

[89] Lee A.K., Potts P.R. (2017). A Comprehensive Guide to the MAGE Family of Ubiquitin Ligases. J. Mol. Biol..

[90] Wüst H.M., Wegener A., Fröb F., Hartwig A.C., Wegwitz F., Kari V., Schimmel M., Tamm E.R., Johnsen S.A., Wegner M. (2020). Egr2-Guided Histone H2B Monoubiquitination Is Required for Peripheral Nervous System Myelination. Nucleic Acids Res..

[91] Xie W., Miehe M., Laufer S., Johnsen S.A. (2020). The H2B Ubiquitin-Protein Ligase RNF40 Is Required for Somatic Cell Reprogramming. Cell Death Dis..

[92] Karpiuk O., Najafova Z., Kramer F., Hennion M., Galonska C., König A., Snaidero N., Vogel T., Shchebet A., Begus-Nahrmann Y. (2012). The Histone H2B Monoubiquitination Regulatory Pathway Is Required for Differentiation of Multipotent Stem Cells. Mol. Cell.

[93] Hardy R.J., Lazzarini R.A., Colman D.R., Friedrich V.L. (1996). Cytoplasmic and Nuclear Localization of Myelin Basic Proteins Reveals Heterogeneity among Oligodendrocytes. J. Neurosci. Res..

[94] Clausell J., Happel N., Hale T.K., Doenecke D., Beato M. (2009). Histone H1 Subtypes Differentially Modulate Chromatin Condensation without Preventing ATP-Dependent Remodeling by SWI/SNF or NURF. PLoS ONE.

[95] Rodriguez P., Munroe D., Prawitt D., Chu L.L., Bric E., Kim J., Reid L.H., Davies C., Nakagama H., Loebbert R. (1997). Functional Characterization of Human Nucleosome Assembly Protein-2 (NAP1L4) Suggests a Role as a Histone Chaperone. Genomics.

[96] Serlidaki D., van Waarde M.A.W.H., Rohland L., Wentink A.S., Dekker S.L., Kamphuis M.J., Boertien J.M., Brunsting J.F., Nillegoda N.B., Bukau B. (2020). Functional Diversity between HSP70 Paralogs Caused by Variable Interactions with Specific Co-Chaperones. J. Biol. Chem..

[97] Aquino D.A., Peng D., Lopez C., Farooq M. (1998). The Constitutive Heat Shock Protein-70 Is Required for Optimal Expression of Myelin Basic Protein during Differentiation of Oligodendrocytes. Neurochem. Res..

[98] López D.J., Rodríguez J.A., Bañuelos S. (2020). Nucleophosmin, a Multifunctional Nucleolar Organizer with a Role in DNA Repair. Biochim. Biophys. Acta.

[99] Frottin F., Schueder F., Tiwary S., Gupta R., Körner R., Schlichthaerle T., Cox J., Jungmann R., Hartl F.U., Hipp M.S. (2019). The Nucleolus Functions as a Phase-Separated Protein Quality Control Compartment. Science.

[100] Mitrea D.M., Cika J.A., Stanley C.B., Nourse A., Onuchic P.L., Banerjee P.R., Phillips A.H., Park C.-G., Deniz A.A., Kriwacki R.W. (2018). Self-Interaction of NPM1 Modulates Multiple Mechanisms of Liquid–Liquid Phase Separation. Nat. Commun..

[101] Zhou H.-X., Nguemaha V., Mazarakos K., Qin S. (2018). Why Do Disordered and Structured Proteins Behave Differently in Phase Separation?. Trends Biochem. Sci..

[102] Rekulapally P., Suresh S.N. (2019). Nucleolus: A Protein Quality Control Compartment. Trends Biochem. Sci..

